# Massive subsurface ice formed by refreezing of ice-shelf melt ponds

**DOI:** 10.1038/ncomms11897

**Published:** 2016-06-10

**Authors:** Bryn Hubbard, Adrian Luckman, David W. Ashmore, Suzanne Bevan, Bernd Kulessa, Peter Kuipers Munneke, Morgane Philippe, Daniela Jansen, Adam Booth, Heidi Sevestre, Jean-Louis Tison, Martin O'Leary, Ian Rutt

**Affiliations:** 1 Centre for Glaciology, Department of Geography and Earth Sciences, Aberystwyth University, Aberystwyth SY23 3DB, UK; 2 Glaciology Group, Department of Geography, Swansea University, Swansea SA2 8PP, UK; 3 Laboratoire de Glaciologie, Département Géosciences, Environnement et Société, Université Libre de Bruxelles, Bruxelles 1050, Belgium; 4 Alfred Wegener Institut, Helmholtz-Zentrum für Polar- und Meeresforschung D-27568, Bremerhaven, Germany; 5 School of Earth and Environment, University of Leeds, Leeds LS2 9JT, UK; 6 Department of Arctic Geology, University Centre in Svalbard, N-9171 Longyearbyen, Norway

## Abstract

Surface melt ponds form intermittently on several Antarctic ice shelves. Although implicated in ice-shelf break up, the consequences of such ponding for ice formation and ice-shelf structure have not been evaluated. Here we report the discovery of a massive subsurface ice layer, at least 16 km across, several kilometres long and tens of metres deep, located in an area of intense melting and intermittent ponding on Larsen C Ice Shelf, Antarctica. We combine borehole optical televiewer logging and radar measurements with remote sensing and firn modelling to investigate the layer, found to be ∼10 °C warmer and ∼170 kg m^−3^ denser than anticipated in the absence of ponding and hitherto used in models of ice-shelf fracture and flow. Surface ponding and ice layers such as the one we report are likely to form on a wider range of Antarctic ice shelves in response to climatic warming in forthcoming decades.

## Abstract (web summary)

The influence of surface ponding on the interior of ice shelves is currently unknown. Here, the authors combine surface and borehole geophysics on the Larsen C Ice Shelf, Antarctica, with remote sensing and modelling and show how pond refreezing increases ice shelf density and temperature.

## Graphical abstract

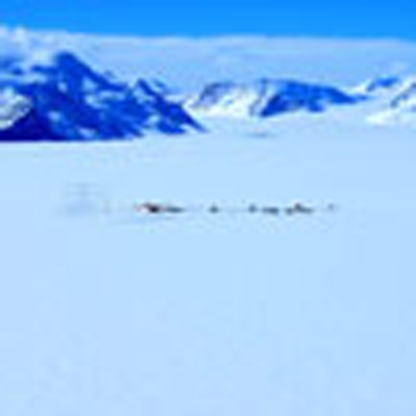

The ∼49,000 km^2^ Larsen C Ice Shelf (LCIS) is considered susceptible to future collapse because of its exposure to intense surface melting^1^ across the northern sector of the Antarctic Peninsula (Fig. 1). Satellite and airborne radar data on the LCIS indicate low firn-air content^2^, promoting the formation of melt ponds and the potential for hydrofracturing^3^,^4^. Analysis of remotely sensed images of Cabinet Inlet, located in the northwest sector of the LCIS, reveals the presence during some summer months of surface melt ponds that generally form in flow-parallel troughs that are some tens of kilometres long and hundreds of metres wide (Fig. 1). These ponds form in Cabinet Inlet as a result of melting by föhn winds that blow from the Graham Land mountains and eastwards through the shelf's northernmost-fringing inlets^5^,^6^,^7^. Although appearing to generate substantial surface melt, these winds do not persist for more than a few days even through the summer, and ponds appear intermittently on satellite images (Fig. 1). The additional stress and hydrofracturing potential of such ponded surface water has been proposed as a means of shelf destabilization^8^,^9^,^10^ and has been implicated in the collapse of Larsen B Ice Shelf in 2002^11^,^12^. However, the implications of such ponding for the formation of new ice and its influence on ice-shelf structure have not been reported.

In this study, we report the presence of a massive ice layer, at least 16 km across, several kilometres long and tens of metres deep, present beneath an area of intermittent pond formation on LCIS, Antarctica. We combine field data with firn-modelling and remotely sensed data to investigate the layer's properties and formation. The layer is found to be composed of two units: an upper, solid ice unit formed largely from the continual refreezing of ponded water; and a lower, infiltration ice unit formed largely from the refreezing of meltwater that has percolated into very dense firn. The layer is found to be ∼10 °C warmer and ∼170 kg m^−3^ denser than that which would have been present in the absence of the influence of intense surface melting and pond formation. The implications of the layer's presence for ice-shelf thickness estimates, flow and stability are explored.

## Results

### Borehole drilling and logging

**Figure 1:**
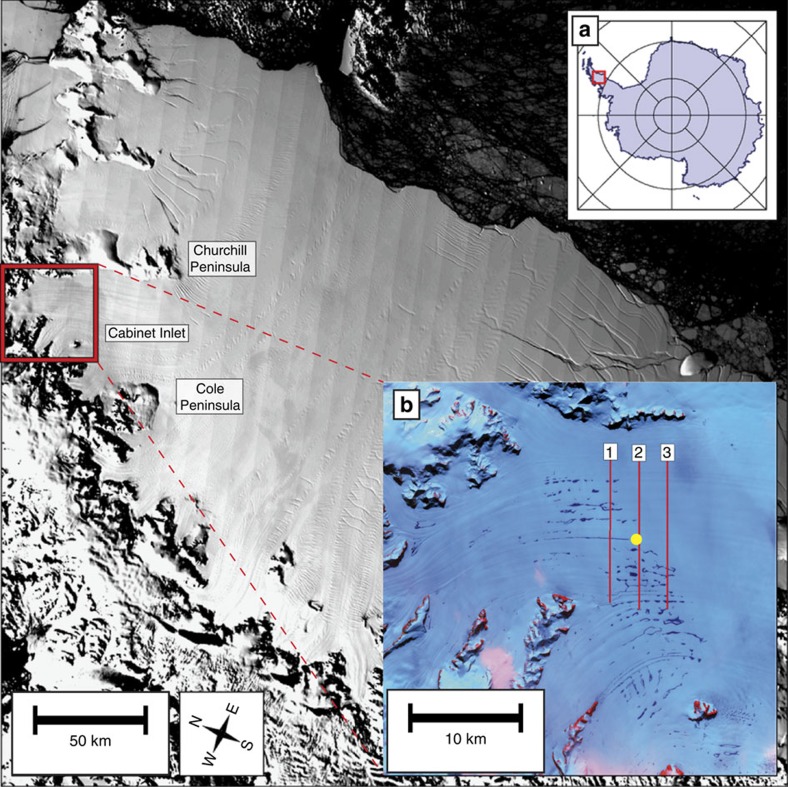
Cabinet Inlet basemap and surface ponding. Location map of Cabinet Inlet study site on Larsen C Ice Shelf based on MODIS data from 3rd December 2014. (**a**) Main figure location in Antarctica (red box). (**b**) Landsat expansion of Cabinet Inlet from 31st December 2001, showing surface ponding (dark patches) and the locations of the borehole (yellow dot) and 200-MHz GPR transects (red lines) presented in Fig. 4.

**Figure 2:**
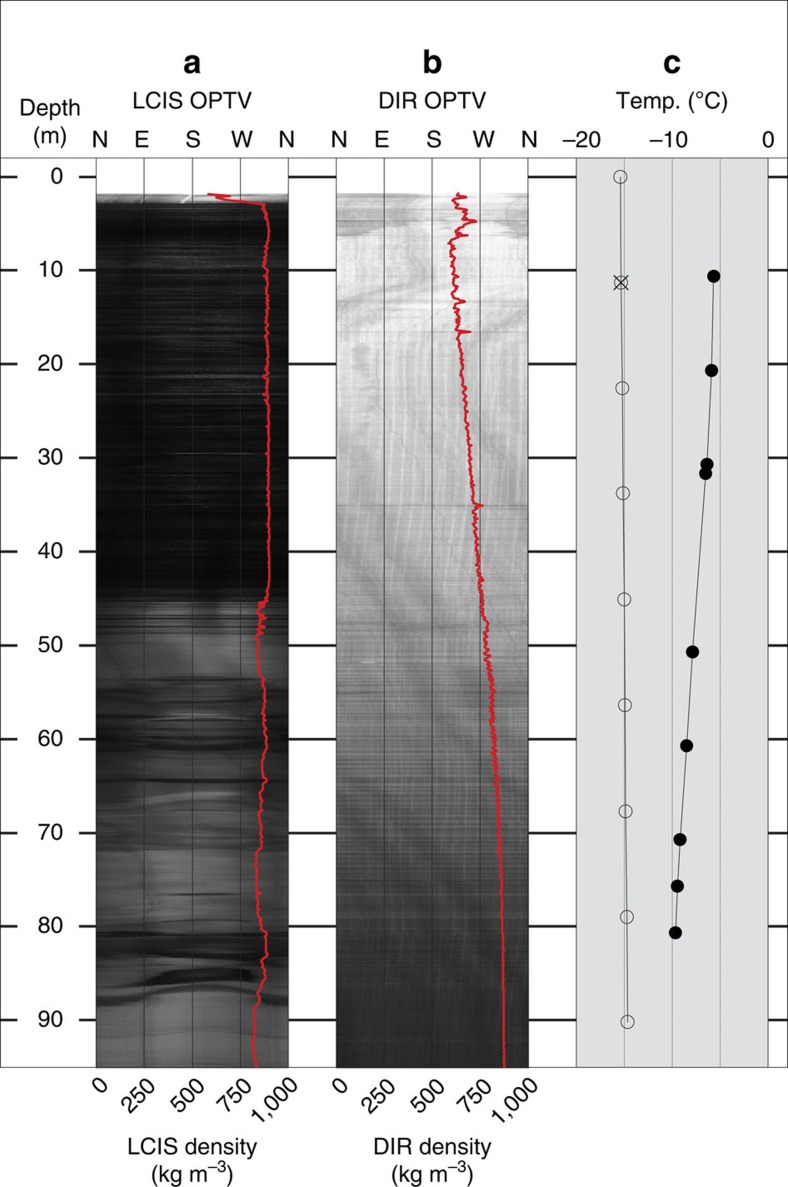
Cabinet Inlet borehole data. (**a**) OPTV log of the LCIS borehole. This raw log is of the complete borehole wall, unrolled to progress north—east—south—west—north from left to right. The log is classified into two units, described and interpreted in the text: Unit 1 extends from a depth of ∼2.9–45 m, and Unit 2 from a depth of ∼45 m to the base. (**b**) OPTV log of a typical ice-shelf borehole unaffected by significant melting (Derwael Ice Rise, Roi Baudouin Ice Shelf, Antarctica) showing (**a**) the normal reduction in luminosity with depth, interpreted as progressive densification from snow, through firn to ice, and (**b**) the usual presence of regular horizontal planes closing up with depth, interpreted as annual layering. Density profiles derived from OPTV luminosity (described in Methods) are superimposed as red lines on both OPTV logs. (**c**) Englacial temperatures (**a**) measured by borehole thermistor string (solid dots), (**b**) that would normally be used in an ice-shelf model for this site (open circles), and (**c**) predicted by our firn model at a depth of 11 m (cross).

In early austral summer 2014/2015, we drilled a ∼100-m long borehole into the flank of a Cabinet Inlet trough indicated by satellite imagery to have repeatedly hosted melt ponds over the past 15 years, but not since 2008/2009. Although the shelf was snow-covered at the time of drilling, inspection of the wall of a 2-m deep pit revealed the presence of both numerous ice layers within the snowpack and an unusually thick ice layer at a depth of 2.0 m that prevented continued excavation. The borehole was logged to a depth of ∼97 m by optical televiewer (OPTV)^13^, providing a geometrically accurate image of the complete borehole wall at a vertical and lateral resolution of ∼1 mm. The resulting OPTV log (Fig. 2a) contrasts starkly with those retrieved from other accumulating ice-shelf and ice-sheet locations (for example, Fig. 2b), including the interior of the Greenland Ice Sheet^14^ and an East Antarctic ice shelf and ice rise^15^,^16^. In each of these other cases, OPTV log luminosity decreases gradually with depth as surface snow metamorphoses through firn to dense ice over depths of several tens of metres. In contrast, our Cabinet Inlet OPTV log (Fig. 2a) shows a sharp contact between high-luminosity surface snow or firn and low-luminosity ice at a depth of only 2.9 m below the ice-shelf surface. This ice extends to the 97-m deep base of the log. Converting the luminosity of this OPTV log to density reveals a mean density for this entire ice layer of 870 kg m^−3^. Figure 2a also reveals a transition in ice type at a depth of ∼45 m, with the overlying layer (named Unit 1) hosting more frequent horizontal layers and being more dense (mean=888 kg m^−3^) than the underlying ice layer (Unit 2; mean=854 kg m^−3^), which is principally composed of bubbly host ice containing coarse, irregularly dipping bubble-free ice layers. The proximity of this generally massive ice layer to the shelf surface, and the sharpness of its contact with the overlying snowpack, preclude formation by the usual process of compaction–metamorphism. Instead, we interpret this Unit 1 ice as having formed as a consequence of refreezing, following periods of intense surface melting and intermittent pond formation. The characteristics revealed by the OPTV image are consistent with this unit (2.9 to ∼45 m) being largely bubble-free pond ice formed from the refreezing of surface water ponded during extended periods of intense, presumably summer, melting. Here the fine-scale horizontal layering apparent in Fig. 2a likely reflects the episodic nature of the process, which would involve four general stages: (a) snow accumulation, (b) surface melting, (c) meltwater infiltration into underlying snow, eventually resulting in its saturation and pond formation, and (d) the freezing of that meltwater layer to form pond ice, probably during early austral autumn. In contrast, Unit 2 (approximately 45–97-m depth) also contains layers of bubble-poor ice, but in this zone they are typically decimetres thick, contorted and isolated within host ice that is optically brighter (likely due to the presence of reflective bubbles; Fig. 2a). We interpret this lower unit as ice dominated by infiltration refreezing formed by meltwater percolating into underlying firn. This still involves the influence of intense surface melting but, in contrast to the Unit 1, of generally insufficient magnitude to form a continuous quasi-massive layer of largely bubble-free pond ice. These physical characteristics and depth ranges of the two units we identify are consistent with the upper (Unit 1) ‘pond ice' forming within the Cabinet Inlet region of pond formation and the lower (Unit 2) ‘infiltration ice' forming, and being inherited from, up-flow of the region of pond formation (Fig. 1).

### Firn modelling and satellite image analysis

**Figure 3:**
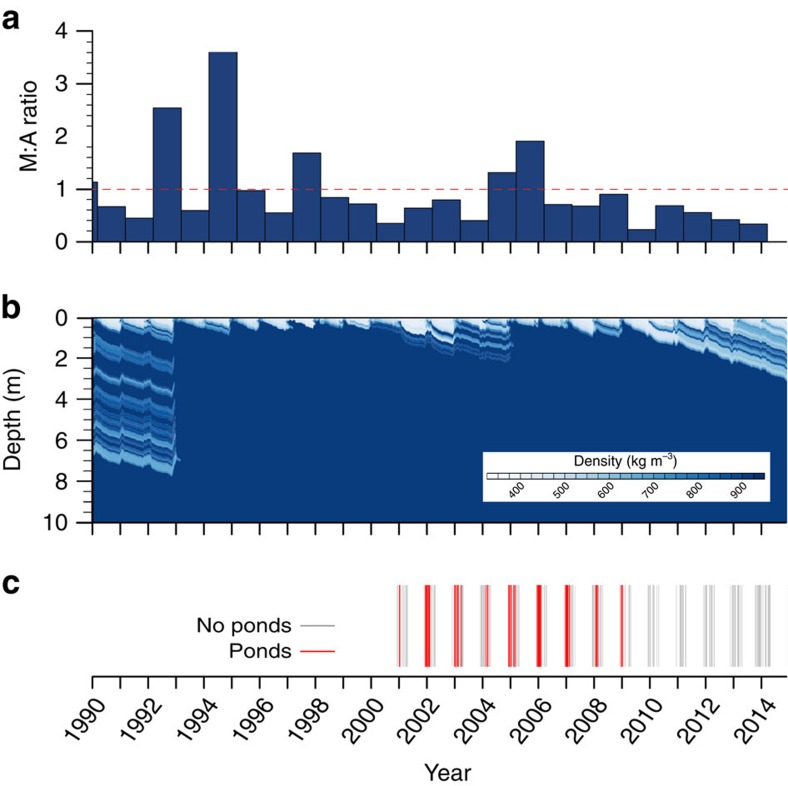
Firn model and remotely sensed outputs for Cabinet Inlet for the period 1st January 1990 to the time of fieldwork on 1 st December 2014. (**a**) Ratio of melt to accumulation (M:A) predicted by the firn model for each 12-month period (1st March–28th February). The horizontal dashed line marks an M:A ratio of 1. (**b**) Predicted firn density plotted against depth. The predicted massive shallow ice layer (dark blue) is never more than ∼8 m below the shelf surface and extends to within ∼1 m of the surface in years 1993–2001 and 2005–2009, both containing individual years of high M:A ratio. (**c**) Classification of time series of summer MODIS satellite images (available since 2001) of Cabinet Inlet according to the presence (red bars) or absence (grey bars) of surface ponds.

**Figure 4:**
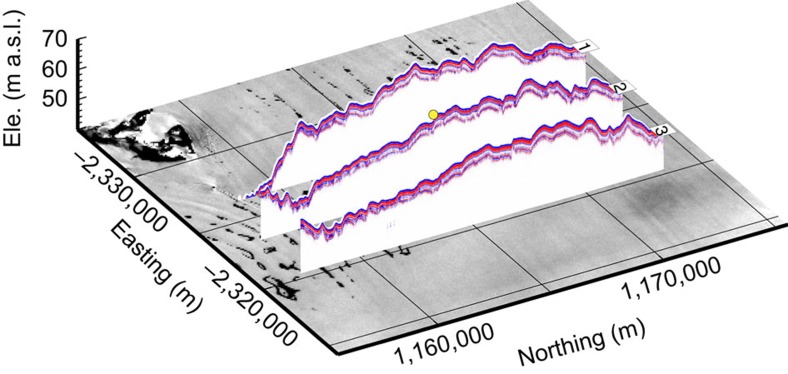
Cabinet Inlet radargrams. 200MHz GPR profiles along the three transects identified in Fig. 1, overlaid on a Landsat image of the region from 31st December 2001 when surface melt ponds were present. The borehole location is marked by the yellow dot.

We evaluate this hypothesis for the recent past through a one-dimensional firn densification and hydrology model^17^, driven by surface mass fluxes and temperature data from the RACMO2.3 regional climate model for Cabinet Inlet^18^. Model results (Fig. 3a,b) indicate the occurrence of intermittent, but substantial, surface melt events at the borehole location, consistent with Unit 1 forming from the refreezing of surface meltwater. As well as predicting the presence of such a layer, the model predicts (Fig. 3b) that the layer's upper surface was, in summer 2014–2015, expected to be 2.9-m below the snow surface, and that the overlying snowpack contains a substantial ice layer between ∼1.9 and 2.2 m, agreeing with our direct observations from snow-pit digging, the OPTV log (Fig. 2a) and ground-penetrating radar (GPR) data (Fig. 4) addressed below. Further, this analysis is supported by a time series of moderate resolution imaging spectroradiometer (MODIS) satellite images (Fig. 3c), indicating that melt ponds formed annually between 2001 and 2009, while none appears in any of the images available between early 2009 and the time of fieldwork in late 2014. This reconstruction matches very closely the RACMO-based firn densification reconstruction (Fig. 3b) with ‘pond years', coinciding with periods of intense near-surface firn densification (for example, 2005–2007).

### Ground-penetrating radar

An approximation of the lateral extent of the massive subsurface ice layer we report is provided by the area known from satellite images to host melt ponds: ∼60-km across-flow and ∼20-km along-flow (Fig. 1). However, the precise degree to which this zone is underlain by massive pond and/or infiltration ice, and the depth of such layers where they are present beyond our borehole, cannot be determined from temporally discrete satellite images. To evaluate this we carried out GPR profiling at 200 MHz along three transects focused on the borehole location (Fig. 1). The resulting radargrams (Fig. 4) reveal the presence of numerous near-surface reflectors and one substantial reflector at a depth that varies between ∼1 and ∼3 m along the transects. Very little radar energy above the background noise level was received from below this reflection. This absence of signal return is consistent with the presence of a refrozen ice layer that, although characterized by minor density stratification, is physically and chemically uniform compared with firn layering unmodified by meltwater. This main reflector is ∼2.9-m deep at the borehole location, coincident with field-based digging, drilling and OPTV logging, as well as with firn modelling (above). We therefore infer that this strong GPR reflection represents the upper surface of a spatially extensive ice layer that is both thick and relatively homogeneous. Since meltwater ponds are confined to surface troughs that persist in MODIS images for decades within this region of Cabinet Inlet, it is unlikely that this ice layer is ubiquitously composed of Unit 1 pond ice. Away from the troughs, this near-surface reflector therefore probably indicates the uppermost surface of an ice layer that is more similar to Unit 2 infiltration ice, that is, still influenced by intense surface melting and subsurface refreezing, but in these areas not actually forming standing ponds. In the absence of further direct investigation, such as by ice coring or borehole analysis, it is not yet possible to determine at high resolution the lateral variability of the two units we identify. Nonetheless, our OPTV and GPR data together indicate that a widespread ice layer extends at least across all of our ∼16-km flow-orthogonal and ∼6-km flow-parallel GPR study area. Our borehole OPTV log also indicates that Units 1 and 2 combined extend to a depth of at least 97 m at the location of our borehole. While it is highly likely that this thickness—defined by the depth of the base of Unit 2—varies spatially across Cabinet Inlet, it is not currently possible to specify this distribution without further borehole and/or GPR data.

## Discussion

The presence of the refrozen ice layer we report above affects the physical properties of the LCIS in at least two ways. First, the layer's density is substantially higher than that of the snow and firn that would otherwise have formed over this depth range by standard compaction–metamorphism. For example, a recent LCIS model^19^ used a density of ∼700 kg m^−3^ for the shelf's uppermost ∼100 m, based on inverting seismic data recorded in the shelf's southern sector. In contrast, our OPTV-derived densities indicate a measured density of ∼870 kg m^−3^ over this depth range in the Cabinet Inlet area, 24% higher. Such a density enhancement influences calculations of the shelf's thickness based on the surface elevation data^20^. In this case, using an ice density of 917 kg m^−3^, a sea water density of 1,026 kg m^−3^, a surface elevation of 63.5 m (Fig. 4) and the assumed firn density of 700 kg m^−3^ for the uppermost 97 m of the shelf yields a total shelf thickness of 382 m. Repeating the calculation with our OPTV-reconstructed density of 870 kg m^3^ for the uppermost 97 m yields a total shelf thickness of 551 m. Although substantially thicker than that calculated from the ‘standard firn' model, a thickness of 551 m is close to that of 564 m reconstructed for the area by the Bedmap2 consortium^21^. This close correspondence reflects the fact that the (altimetry-based) Bedmap reconstructions include a correction to account for enhanced densification, resulting from active surface melting on ice shelves^22^. Our OPTV-based density reconstruction also yields a firn-air content (the column-length equivalent accounted for by material with a density lower than that of bubble-free glacial ice) of 5.0 m. While this value is substantially lower than the 23.0 m that would result from firn of density 700 kg m^−3^, it is far closer to the spatially distributed range of 0–4 m predicted for the area based on recent combined analyses of remotely sensed surface elevation and shelf thickness fields^9^,^10^. However, direct comparisons such as these are somewhat confounded by our firn-air content of 5.0 m being based on a single-point measurement (recorded, as noted above, on the limb of an elongate trough hosting an ephemeral surface pond), whereas the reconstructions based on remotely sensed data integrate data over a coarser spatial field.

Second, as well as altering its density, the ice column will be warmed by latent heat released by the freezing of ponded and percolating meltwater. This effect is quantified by a thermistor string installed into the borehole following OPTV logging (Fig. 2c). The mean annual temperature measured at a depth of 11 m was −5.9 °C, while the mean annual surface temperature at the site (which normally defines that at a depth of 10–15 m in the absence of significant refreezing) is −16.9 °C. Further, the entire ice profile recorded by our thermistor string (Fig. 2c) is warmer than would be expected without factoring in refreezing of ponded meltwater. This effect is confirmed by our firn model, which under-predicts englacial temperatures despite accounting for heat released by the refreezing of percolating meltwater. In this case, modelled pore-water refreezing contributes ∼1.5 °C to the firn, yielding a predicted temperature at 11 m of −15.4 °C (Fig. 2c). This is still almost 10 °C colder than our measured firn temperature at that depth, demonstrating that the refreezing of ponded meltwater (not included in the model) provides significant, hitherto unconsidered, englacial heating in this region. As well as being denser, this ice is therefore also warmer than that which would otherwise be used in numerical models of the flow of the LCIS^19^.

Replacing on the order of 10 × 10 × 0.1 km of standard firn with relatively warm and dense ice will exert some influence on ice shelf flow and stability. However, evaluating this influence at the ice-shelf scale is not straightforward. In the first instance, the warmer ice layer we identify will be less viscous than colder ice, and this may go some way to accounting for the anomalously high-rate factor that has in the past been necessary for numerical models of the flow of the LCIS, and in particular its northern sector, to match empirical data^23^,^24^. A consequence of such a temperature-induced acceleration would be a general reduction in back stress within confined embayments, such as Cabinet Inlet, potentially increasing shear stresses along the flow unit's lateral margins. This process is consistent with observations that the northern sector of Larsen B Ice Shelf experienced an increase in lateral rifting before its break up in 2002 (ref. 25). In contrast, the influence of the layer's presence on brittle deformation may be in the opposite direction, with enhanced ductile flow accommodating strain and the solid ice we report being more resistant to tensile fracture than lower-density and finer-grained firn. In such cases, the flow-parallel alignment of elongate solid ice bodies such as that we report herein might serve to resist the flow-orthogonal crevassing that commonly develops in response to longitudinal tensile stresses, approaching the marine limit of ice shelves. Finally, the spatial extent of this layer at the ice-shelf scale, and the way in which its deformation interacts with that of other material units, such as suture ice, and basal channels and crevasses, will also influence the way in which the layer's presence affects overall shelf stability. In particular, longitudinal troughs located on the surface of ice shelves have been related to spatially coincident basal channels^26^, which have themselves been associated with shelf instability^27^ through local thinning and crevassing^28^,^29^. Although it is not yet known whether the surface troughs on LCIS investigated herein are associated with such basal channels, the recent finding that material density is locally enhanced below similar surface troughs on the Roi Baudouin Ice Shelf, Antarctica^30^, is consistent with the enhanced melting and massive ice formation reported herein. In the light of these complexities, identifying and exploring the net influence of pond ice formation on ice-shelf stability can only be achieved with confidence by including the full spatial extent and physical properties of the refrozen ice layer into a shelf-wide flow and fracture model, which may be regarded as a future research priority.

The surface ponding responsible for the subsurface ice layer we report herein is currently restricted to warmer regions of Antarctica's fringing ice shelves and in particular, but not exclusively, to the northern and western sectors of the Antarctic Peninsula. However, regional warming is predicted to spread southwards and intensify substantially over forthcoming decades^5^,^31^, so ponding is expected also to become more widespread. Similarly, massive layers of warm, dense ice are therefore highly likely to form within ice shelves present across a substantial area of Antarctica, perhaps the entire continent if warming continues into the 22nd century^5^, with important consequences for ice-shelf flow, hydrofracture and stability.

## Methods

### Borehole drilling and logging

**Figure 5:**
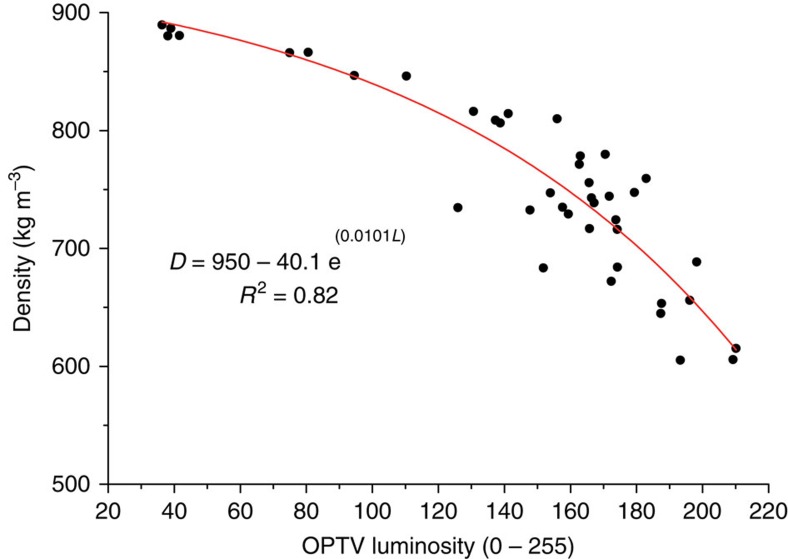
Optical televiewer density–luminosity calibration. Material density, measured gravimetrically on core samples, plotted against equivalent OPTV luminosity, both measured along a borehole located on Roi Baudouin Ice Shelf, East Antarctica.

The borehole was drilled by pressurized hot water and logged by OPTV^13^. The resulting OPTV log, analysed by WellCAD and BIFAT software^32^, provides a geometrically accurate image, with a pixel size of ∼1 mm, of the material composition of the complete borehole wall, as well as the structural geometry of layers and inclusions intersecting it^33^. Since the OPTV log records an image of reflected light, it also provides a proxy for the density of compacted snow, firn and ice due to the progressive decrease in reflectivity, as voids close and bubbles are occluded and collapsed^13^,^15^. Hubbard *et al*.^15^ exploited this relationship and identified an exponential relationship between OPTV luminosity (*L*) and material density (*D*) on the basis of samples recovered from a core retrieved from a borehole logged by OPTV on the Roi Baudouin Ice Shelf, East Antarctica. However, due to equipment loss, the light-emitting diode brightness of the OPTV probe used in the current study was different from that of Hubbard *et al*.^15^, and a new calibration was undertaken. This was based on the correlation of 40 core samples recovered from a logged borehole, again located on the Roi Baudouin Ice Shelf (Fig. 5). The new calibration yields a best-fit regression equation of *D*=950–40.1 *e*^(0.0101*L*)^ (*R*^2^=0.82), with root mean square values of the residuals of 40.4, 35.2 and 21.7 kg m^−3^ for the density ranges 600–700, 700–800 and 800–900 kg m^−3^, respectively. While these values provide an error range for absolute densities derived from OPTV luminosity, the relative changes in density reported herein, that is, along a single borehole log, reduce to the precision of the method. This is approximated by the density range (910 kg m^−3^) divided by the luminosity range (256), or ∼3.5 kg m^−3^.

Borehole temperatures (Fig. 2c) were recorded by negative temperature coefficient thermistors, recorded across a Wheatstone half-bridge by Campbell Scientific micro-loggers. Resistances were converted to temperature using a polynomial^34^ fitted to the manufacturer's calibration curve refined via a second-stage calibration in a distilled water/ice bath. Once recalibrated, sensors were replaced into a new water/ice bath to determine temperature error, yielding a root mean square error of ±0.03 °C. Borehole temperatures were logged for at least 100 days, and the undisturbed ice temperature was calculated from an exponential function fitted to the cooling curve^35^. By this time, all temperatures recorded below the near-surface zone of seasonal thermal disturbance had stabilized to values that varied (over timescales of days to weeks) by <0.05 °C. Thus, the englacial temperatures we report herein were not influenced by the hot-water drilling process.

### Firn modelling

The firn model used is IMAU-FDM v1.0, which takes into account firn compaction, meltwater percolation and refreezing^17^,^18^. At the surface, the firn model is forced with mass fluxes (snowfall, snowmelt, rain, sublimation, and snowdrift sublimation and erosion) and surface temperature from the regional climate model RACMO2.3, run at a 5.5-km horizontal resolution in a domain over the Antarctic Peninsula.

### Satellite image analysis

MODIS Terra and Aqua level 1 (250 m) data were ordered via the level 1 and atmosphere archive and distribution system, and used to check for the presence of melt ponds in Cabinet Inlet. A total of 577 cloud-free band 2 (848 nm) images between 1^st^ December and 30th April from 2000 (Aqua) or 2002 (Terra) to 2015 were classified to generate the time series shown in Fig. 3c.

### Ground-penetrating radar

GPR data (Fig. 4) were collected using a Sensors and Software Pulse Ekko Pro system operated in common offset mode with a 0.8-ns sample interval and a 4,000-ns sample window. The console was mounted on the skidoo and the 200 MHz antennae towed 15 m behind on a plastic sledge at ∼10 km h^−1^ with an eight stack trace recorded every 3 m. A Leica VIVA GS10 GNSS rover unit was connected directly to the GPR console; the base station was located at the drill site. Surface elevation was referenced to the Earth Gravitational Model 1996 geoid, taken to represent sea level, and no correction was made for dynamic topography. GPR data were processed in Reflex-W. Processing steps included de-wow of 25-ns filter length, spherical divergence compensation, spectral whitening between 70 and 200 MHz, and a two-dimensional mean-averaging filter. The static correction was based on Leica Geo Office post-processed GNSS data, resulting in a vertical accuracy of ±0.5 m. Radar wave propagation velocities used to convert travel times to the depths shown in Fig. 4 were 0.2 m ns^−1^ for the uppermost 3 m (based on a local 500 MHz common midpoint gather from the uppermost reflector of Unit 1) and a typical value for glacial ice^36^ of 0.17 m ns^−1^ below that.

### Data availability

The data that support the findings of this study are available from http://www.projectmidas.org/data/hubbard2016/ and from the corresponding author upon request.

## Additional information

**How to cite this article**: Hubbard, B. *et al*. Massive subsurface ice formed by refreezing of ice-shelf melt ponds. *Nat. Commun.* 7:11897 doi: 10.1038/ncomms11897 (2016).
